# Genetic Variation and Biological Activity of Two Closely Related Alphabaculoviruses during Serial Passage in Permissive and Semi-Permissive Heterologous Hosts

**DOI:** 10.3390/v11070660

**Published:** 2019-07-18

**Authors:** Isabel M. Belda, Inés Beperet, Trevor Williams, Primitivo Caballero

**Affiliations:** 1 Institute for Multidisciplinary Research in Applied Biology, Universidad Pública de Navarra, 31006 Pamplona, Navarra, Spain; 2 Departamento de Investigación y Desarrollo, Bioinsectis SL, Pol. Ind. Mocholi Plaza Cein 5, Nave A14, 31110 Noain, Navarra, Spain; 3 Instituto de Ecología AC, Xalapa, 91073 Veracruz, Mexico

**Keywords:** host range, *Spodoptera littoralis*, *Spodoptera exigua*, genetic stability, insecticidal characteristics

## Abstract

Phylogenetic analyses suggest that Mamestra brassicae multiple nucleopolyhedrovirus (MbMNPV) and Helicoverpa armigera multiple nucleopolyhedrovirus (HearMNPV) may be strains of the same virus species. Most of the studies comparing their biological activities have been performed in their homologous hosts. A comparison of host range and stability in alternative hosts was performed. The host range of these viruses was compared using high concentrations of inoculum to inoculate second instars of six species of Lepidoptera. One semi-permissive host (*Spodoptera littoralis*) and one permissive host (*S. exigua*) were then selected and used to perform six serial passages involving a concentration corresponding to the ~25% lethal concentration for both viruses. Restriction endonuclease analysis showed fragment length polymorphisms in every host-virus system studied. In *S. littoralis*, serial passage of MbMNPV resulted in decreased pathogenicity and an increase in speed-of-kill, whereas no significant changes were detected for HearMNPV with respect to the initial inoculum. In contrast, both viruses showed a similar trend in *S. exigua*. These results highlight the low genetic diversity and a high phenotypic stability of HearMNPV with respect to the original inoculum after six successive passages in both insect hosts. This study concludes that host-baculovirus interactions during serial passage are complex and the process of adaptation to a novel semi-permissive host is far from predictable.

## 1. Introduction

The family *Baculoviridae* comprises double-stranded DNA viruses that are specific to invertebrate hosts. Their insecticidal properties combined with high host specificity mean that they can be valuable agents for the biological control of insect pests of field and greenhouse crops and in forest ecosystems [1,2]. Baculoviruses belonging to the genus *Alphabaculovirus* (lepidopteran-specific nucleopolyhedroviruses, NPV) show important variation in their host range, from viruses that are highly host specific, such as Lymantria dispar multiple nucleopolyhedrovirus (LdMNPV), to viruses that can productively infect multiple species of hosts from different lepidopteran families, such as Autographa californica multiple nucleopolyhedrovirus (AcMNPV) [3].

Baculovirus classification based on the host from which the virus was isolated has obvious drawbacks for viruses that can productively infect multiple species of hosts. This can also create confusion when several baculoviruses are isolated from the same host species. To address this issue, the International Committee on Taxonomy of Viruses (ICTV) determined that the definition of species status should involve phylogenetic criteria for lepidopteran specific baculoviruses, based on the genetic distances of the *lef-8*, *lef-9* and *polh/gran* genes measured by the Kimura 2-parameter (K2P) value [4]. For viruses with intermediate K2P values, additional information is needed to decide whether particular baculoviruses belong to the same species or not. Therefore, information on biological properties and ecological niche can contribute to species definitions for closely-related viruses [5]. For example, according to their genetic distance characteristics, Helicoverpa armigera multiple NPV (HearMNPV), Mamestra brassicae multiple NPV (MbMNPV) and Mamestra configurata NPV-B (MacoMNPV-B) may be considered as strains of the same virus species [4,5,6,7,8,9], although the ICTV currently considers MbMNPV and MacoMNPV-B as different species [10]. Notwithstanding the above, recent alternative approaches based on coalescence theory allow the clustering of sequences into species groups, which may prove particularly valuable for species delimitation in baculoviruses [9]. 

Both MbMNPV and HearMNPV can productively infect a relatively wide range of lepidopteran species [11,12,13]. Biological studies confirm that these viruses share a high degree of similarity [14,15]. However, the biological activity of these viruses has only been quantified and compared in the respective homologous hosts, *M. brassicae* and *H. armigera* [14]. Importantly, the homologous host, which is usually the host species from which the virus was first isolated, is not necessarily the species in which the virus has the highest biological activity [3,13]. In this sense, further studies of host range and biological activities in alternate hosts could provide a better understanding of the biological similarities between MbMNPV and HearMNPV.

Host range in baculoviruses is characterized by high variation in the susceptibility of different hosts to a particular virus [16], but is currently difficult to predict from phylogenetic relationships [3]. A host species that dies from polyhedrosis disease following inoculation with a dose of occlusion bodies (OBs) similar to that of the homologous host of the same developmental stage, is usually classified as a permissive species. In contrast, a host species that requires a much larger inoculum dose to elicit lethal polyhedrosis disease, or in which the virus replicates poorly and OB yields are low, is usually classified as a semi-permissive species [17]. 

Natural baculovirus populations are characterized by high genetic variability that is likely to influence the host range and their ability to adapt to novel hosts [18]. Serial passage experiments have shown that baculoviruses can undergo genetic and phenotypic changes due to genetic bottlenecks and genetic drift when the inoculum is repeatedly used to infect semi-permissive insect hosts and cells [19,20,21,22]. During serial passage, adaptation to the semi-permissive host can involve complex genetic diversification, including alterations in the abundance of particular genotypic variants or the emergence of new genotypes due to recombination events [18,21]. Furthermore, replication in a particular host also affects virus composition, as a proteomic analysis of budded virions (BV) and occlusion derived virions (ODV) of MbMNPV following replication in two different host species has revealed the presence of host-specific proteins associated with virions [23].

In this study, MbMNPV and HearMNPV were selected in order to investigate the degree of similarity of their biological properties in alternative hosts. First, the degree of susceptibility to these viruses was studied in six different lepidopteran pest species. One semi-permissive and one permissive host were then selected for serial passage studies. The genetic variability and biological activity of each virus in the semi-permissive host *Spodoptera littoralis* and in the permissive host *S. exigua* was studied with the aim of identifying the variables (type of host, virus identity or number of passages) that most affect the genetic and phenotypic characteristics that contribute to improving the insecticidal properties of these viruses.

## 2. Materials and Methods 

### 2.1. Insects and Viruses

The larvae of lepidopteran species were obtained from laboratory colonies reared at the Universidad Pública de Navarra (UPNA) and maintained at 25 ± 1 °C, 75% relative humidity and 16 h light: 8 h dark photoperiod on a wheat germ-based semi-synthetic diet [24]. A colony of *S. littoralis* was obtained from Israel, whereas the *S. exigua* colony was provided by Universitat de Valencia, Valencia, Spain. Due to the detection of a SeMNPV infection during serial passage in this species, a new *S. exigua* colony was established from eggs provided by Entomotech (Almería, Spain). These insects were tested for the presence of persistent SeMNPV infections by qPCR before establishing the new colony.

The MbMNPV strain was isolated from the microbial insecticide Mamestrin^®^ (NPP, Nogueres, France). In contrast, HearMNPV, originally from the former USSR [25], was kindly provided by D. Winstanley (Horticulture Research International, Wellesbourne, Warwick, UK). The OBs of each virus were used to orally inoculate fifth instars of the respective homologous hosts, *M. brassicae* and *H. armigera*, at a concentration that resulted in approximately 90% mortality, to obtain the passage zero population of each virus, named MbMNPV-P_0_ and HearMNPV-P_0_.

### 2.2. Host Range Screening of MbMNPV and HearMNPV

In order to compare the host range of each virus, susceptibility tests were performed on six lepidopteran species (*H. armigera*, *Lobesia botrana*, *M. brassicae*, *Ostrinia nubilalis*, *S. exigua* and *S. littoralis*). For each species, groups of 28 newly-molted second instars that had been starved for 12 h were inoculated with a single high concentration (5 × 10^6^ OBs/mL) of MbMNPV OBs or HearMNPV OBs using the droplet feeding method [26]. The larvae that drank OB suspension within 10 min were reared individually at 25 ± 1 °C in cups with semi-synthetic diet and mortality was recorded until larvae had died or pupated. The groups of 28 mock-infected larvae were used as the control. Each test was performed in triplicate using different batches of insects. Host species were classified as non-permissive (0% mortality), semi-permissive (<70% mortality) or permissive (>70% mortality). In order to preclude the activation of a hypothetical latent infection in a host species that experienced virus-induced mortality, the OBs from virus-killed cadavers of each species were re-amplified in fourth instars of the corresponding experimental host. The resulting virus-killed cadavers were homogenized in sterile water and filtered through muslin. The OBs were washed twice with 0.1% sodium dodecyl sulfate (SDS) and twice with double-distilled water and re-suspended in double-distilled water. Genomic DNA was then extracted and subjected to restriction endonuclease analysis to confirm the identity of the virus produced. For this, ODVs were released from 2 × 10^8^ OBs by mixing 20 μL of OB suspension with 50 μL of 0.5 M Na_2_CO_3_, 25 μL of 10% (*w*/*v*) SDS and 155 μL of sterile double-distilled water followed by incubation at 60 °C during 10 min. The undissolved OBs were removed by centrifugation at 2300× *g* for 5 min. The supernatant was treated with 15 μL of proteinase K (20 mg/mL) and incubated at 50 °C for 30 min. Protein was then precipitated by the addition of 150 μL of MPC Protein Precipitation Reagent (Epicentre Technologies Corp., Madison, WI, USA). Viral DNA was isolated by alcohol precipitation. The resulting pellet was re-suspended in 60 μL of 0.1 × TE buffer (10 mM Tris, 1 mM EDTA, pH 8.0). Approximately 1 μg of DNA was digested with 2 µL PstI and HindIII (FastDigest, ThermoFisher) in a total volume of 20 µL at 37 °C for 1 h. The restriction fragments were separated by electrophoresis in 1% agarose gels in TAE buffer (40 mM Tris, 20 mM acetic acid, 1 mM EDTA, pH 8.0) at 16V for 15 h. The fragments were visualized on a UV transiluminator after GelRed® (Biotium) staining.

### 2.3. Serial Passage in S. littoralis and S. exigua

**Figure 1:**
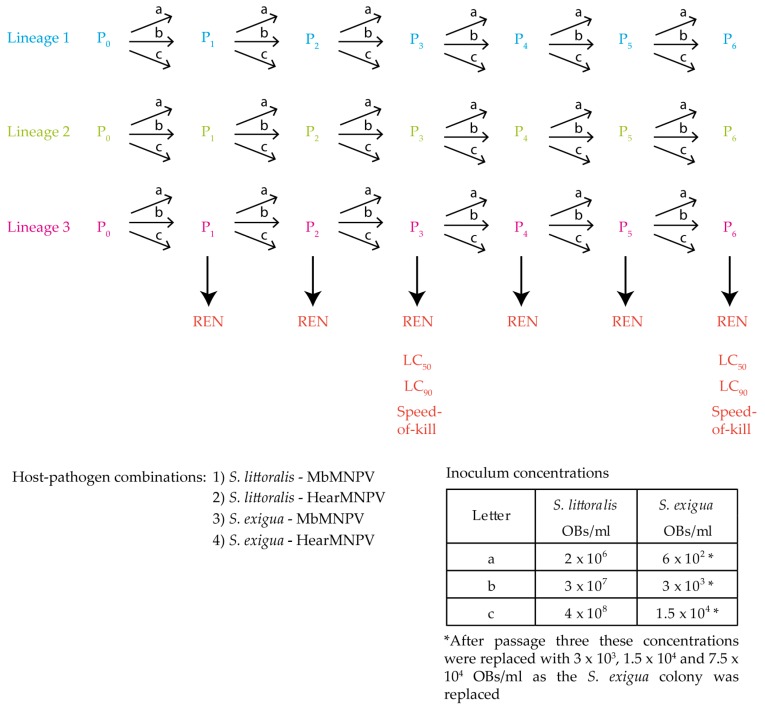
Schematic figure of the serial passage infection process from passage zero to six (P_0_–P_6_). The inoculum concentration (a, b or c) that resulted in ~25% mortality in experimental insects was used as the inoculum for the following passage. Inoculum occlusion bodies were subjected to restriction endonuclease analysis (REN) at every passage. LC_50_ and LC_90_: Lethal concentration 50% and 90%.

For each virus, six steps of serial passage were performed in fourth instars of the semi-permissive host, *S. littoralis,* and the permissive host, *S. exigua* (Figure 1). Fourth instar larvae were selected to ensure the production of relatively large quantities of OBs required for each step of the serial passage experiments. For each host-virus combination, three replicate lineages were performed (lineages 1 - 3). To establish these lineages, for the first passage, the original inoculum of each virus (MbMNPV-P_0_ and HearMNPV-P_0_) was used to infect three batches of larvae of each species. Then, following death by polyhedrosis, dead infected insects of each lineage were pooled, purified and the resulting OBs were used for the next passage. At each passage, three groups of 36 larvae in each lineage were orally inoculated and were reared individually on semi-synthetic diet at 25 ± 1 °C until death or pupation. For every lineage and passage, groups of 28 larvae were mock-infected as controls. The serial passages were performed using a 25% lethal concentration (LC_25_). This was undertaken as low inoculum concentrations are likely to favor the transmission of virus genotypes that are prevalent in the inocula due to efficient replication in the previous heterologous host, and to hinder the transmission of defective genotypes that can arise during serial passage [27]. To ensure a ~25% prevalence of mortality, three inoculum concentrations (labelled a, b and c in Figure 1) were used to infect each group of insects at each passage using the droplet feeding method [26]. The inoculum concentrations were 2 × 10^6^, 2 × 10^7^ and 2 × 10^8^ OBs/mL for both viruses in *S. littoralis* and 6 × 10^2^, 3 × 10^3^ and 1.5 × 10^4^ OBs/mL for both viruses in *S. exigua*. Once the SeMNPV infection was detected in lineage 2 of *S. exigua*-MbMNPV system, the whole *S. exigua* population was replaced with free-virus insects from southern Spain. The new insect colony was subjected to qPCR screening to ensure the absence of persistent SeMNPV infections, as described previously [28]. The new *S. exigua* population was found to be more resistant to infection, and inoculum concentrations were increased to 3 × 10^3^, 1.5 × 10^4^ and 7.5 × 10^4^ OBs/mL for passages four to six. Virus-killed cadavers of the treatment that resulted in ~25% mortality were pooled within each lineage, purified, counted twice using a Neubauer hemocytometer and used as oral inoculum for the next passage. This same methodology was performed for every passage (Figure 1).

### 2.4. Genetic Variation in Viruses during Serial Passage

To study the genetic variation in the viral populations during serial passage in a particular host, restriction endonuclease (REN) analysis of DNA from OBs obtained at each passage was performed and compared with the MbMNPV-P_0_ and HearMNPV-P_0_ initial inoculum. The REN analysis was performed using PstI and HindIII for both viruses as described in Section 2.2. Additionally, when contamination by an unexpected NPV was detected, additional bands present in the REN profile were studied to establish the identity of the contaminant virus. The presence of contaminant viruses was determined by quantitative PCR (qPCR) using specific primers targeted at the *bro-a1* and *se28* genes of MbMNPV and SeMNPV, respectively (Table 1). For this, DNA was extracted as described in Section 2.2, adjusted to a total concentration of 1 ng/μL and quantified by qPCR, as described previously [29].

### 2.5. Insecticidal Properties of Viruses Following Serial Passage

To examine changes in the biological activity of MbMNPV and HearMNPV after serial passage in the selected permissive and semi-permissive hosts, bioassays were performed on the OB suspensions obtained at passages three and six of each host-virus combination and for each lineage to determine OB pathogenicity and speed-of-kill (Figure 1). The OBs used as the initial inoculum (MbMNPV-P_0_ and HearMNPV-P_0_) were used as reference treatments in every bioassay. 

Pathogenicity, expressed as 50% and 90% lethal concentrations (LC_50_ and LC_90_), was estimated in newly-molted second instars of each host species using the droplet feeding method [26]. Further, groups of 28 larvae were inoculated with one of five OB concentrations for *S. exigua* and one of six OB concentrations for *S. littoralis*. The same OB concentrations were used for both viruses in *S. littoralis* (8 × 10^5^, 4 × 10^6^, 2 × 10^7^, 1 × 10^8^, 5 × 10^8^ and 2.5 × 10^9^ OBs/mL) and in *S. exigua* (3 × 10^3^, 1.5 × 10^4^, 7.5 × 10^4^, 3.75 × 10^5^ and 1.88 × 10^6^ OBs/mL). In both cases, the range of concentrations was estimated to kill between 5 and 95% of inoculated insects. The larvae that ingested the inoculum suspension in a 10 min period were individually transferred to 28-well plates containing semi-synthetic diet and were incubated at 25 ± 1 °C. The control insects were treated identically but did not consume OBs. Virus-induced mortality was recorded at 24 h intervals until death or pupation. The bioassays were performed in triplicate using different batches of insects. Virus-induced mortality results were subjected to probit analysis using POLO-PLUS software [30].

To determine the speed-of-kill responses (median time to death), groups of 28 newly-molted second instars of each host species were treated with a single OB concentration estimated to result in ~90% mortality (LC_90_), or a sucrose solution free of OBs as a control treatment. Larvae that drank the inoculum within 10 min were reared individually at 25 ± 1 °C and virus-induced mortality was recorded at 8 h intervals until all the larvae had died or pupated. The experiment was performed on three occasions. The time-mortality data were subjected to Kaplan–Meier survival analysis using SPSS Statistics v.25.0 software [31].

## 3. Results

### 3.1. Host Range Screening

The insect species tested in the host range evaluation were classified as non-permissive, semi-permissive and permissive (Table 2). Semi-permissive species experienced 34–38% mortality, whereas the mortality range observed in the permissive species was 87–100%. The mortality responses were similar for MbMNPV and HearMNPV in all lepidopteran species tested, which resulted in two non-permissive hosts (*L. botrana* and *O. nubilalis*), one semi-permissive host (*S. littoralis*) and three permissive hosts (*H. armigera*, *M. brassicae* and *S. exigua*) (Table 2). As a result, *S. littoralis* was selected as the semi-permissive host for the serial passage experiment, whereas *S. exigua* was selected as the permissive host.

### 3.2. Virus-Induced Mortality Observed during Serial Passage

**Figure 2:**
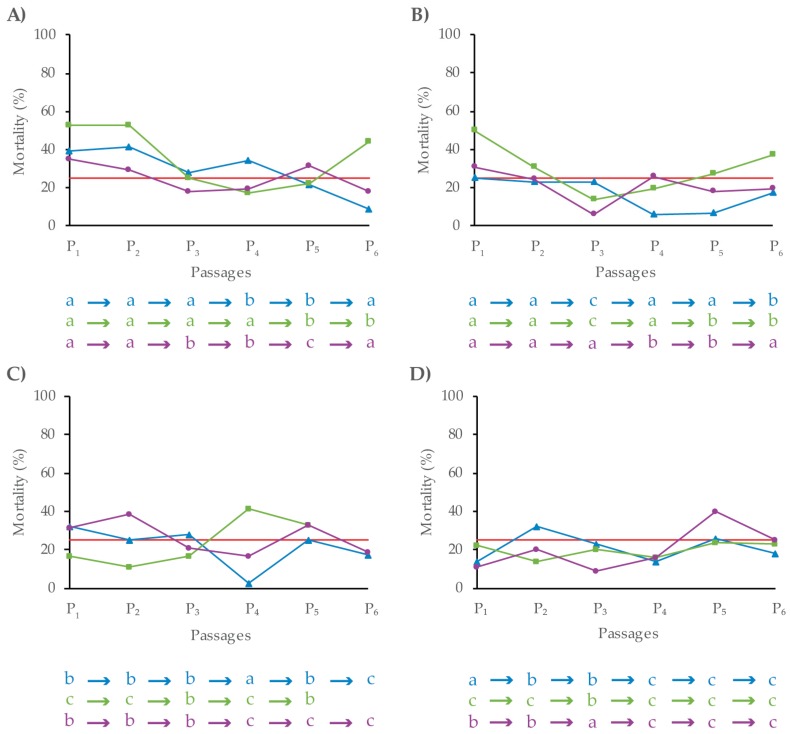
The percentage of mortality recorded in each passage in the three lineages of four host-pathogen systems. (**A**) *S. littoralis* – MbMNPV, (**B**) *S. littoralis* – HearMNPV, (**C**) *S. exigua* – MbMNPV, (**D**) *S. exigua* – HearMNPV. Lineage 1 is indicated in blue, lineage 2 in green and lineage 3 in purple. The horizontal red line indicates 25% mortality. The letters of the different lineages correspond to the three concentrations of inocula used in each passage: (a) low, (b) medium, (c) high.

In every host-virus combination, three different concentrations (shown as a: low, b: medium; c: high in Figure 2) were used for each passage. The inoculum that caused ~25% mortality was selected for the subsequent passage. The inoculum concentration selected in each host-virus combination was not always the same, but varied at different passages and among the three lineages (Figure 2).

### 3.3. Genetic Analysis of OBs Produced during Serial Passage

**Figure 3:**
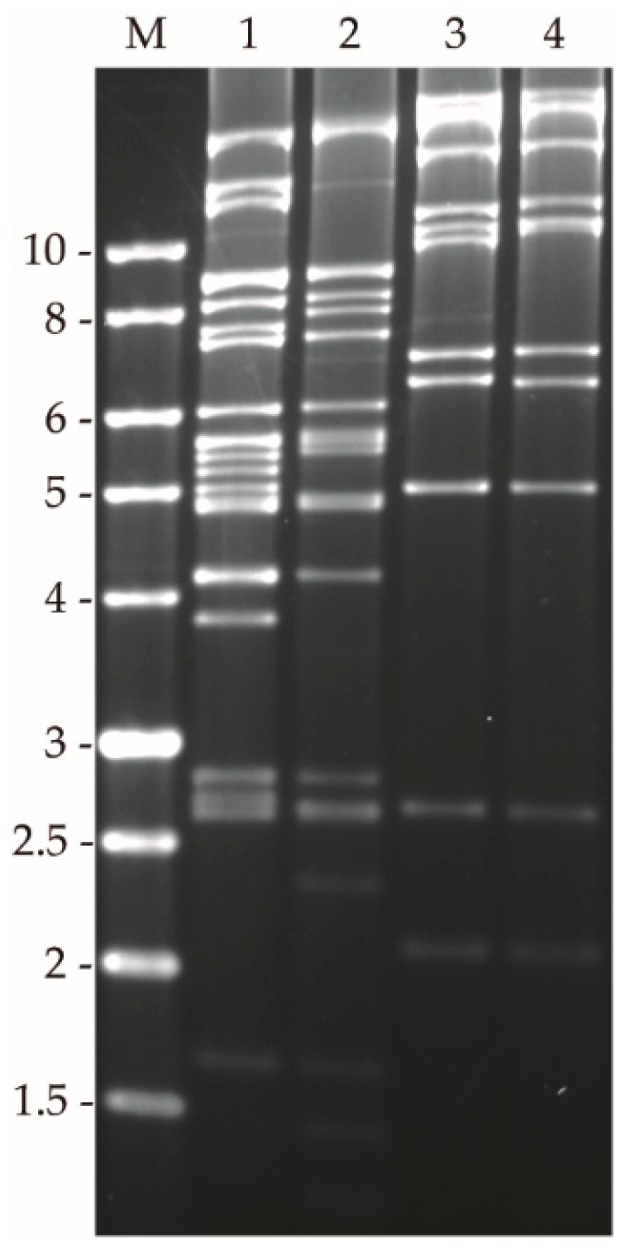
REN profiles of MbMNPV and HearMNPV initial inocula (P_0_). 1–2: HindIII restriction profile; 3–4: PstI restriction profile. 1 and 3: MbMNPV-P_0_; 2 and 4: HearMNPV-P_0_. M indicates 1 Kb molecular size marker (Nippon) and fragment sizes in kilobases (Kb) are indicated on the left.

Restriction endonuclease analysis of the starting inoculum of both viruses (MbMNPV-P_0_ and HearMNPV-P_0_) was performed using PstI and HindIII in order to compare broadly the genetic characteristics of these viruses and to establish the initial identities of the starting inocula (Figure 3). REN profiles showed fragment length polymorphisms that were used for virus identification during serial passage. Variability in restriction fragments was readily detected with PstI in MbMNPV and with HindIII in HearMNPV. Therefore, only one REN profile per virus passage is presented.

#### 3.3.1. Analysis of Viral DNA Produced in *S. littoralis*

**Figure 4:**
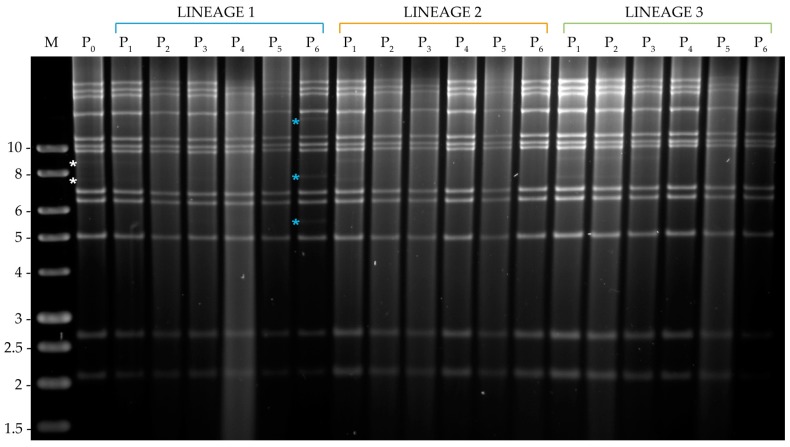
PstI profiles of the genomic DNAs of OBs obtained after serial passage of MbMNPV in the semi-permissive host, *S. littoralis*, from passage zero to six (P_0_–P_6_). M indicates 1 kb molecular size marker (Nippon) and fragment sizes are indicated on the left. White asterisks indicate P_0_ submolar bands. The new restriction fragments with respect to the MbMNPV-P_0_ profile are indicated by blue asterisks.

In MbMNPV profiles, two weak submolar PstI bands were present in MbMNPV-P_0_ with a size of approximately 7.5 kb and 9 kb, both of which gradually disappeared in the three infection lineages during the serial passage and were not visible at the final passage (Figure 4). However, MbMNPV changed only in lineage 1 after passage six with the appearance of three new weak submolar PstI bands of approximately 5.5, 7.6 and 15 kb (Figure 4).

**Figure 5:**
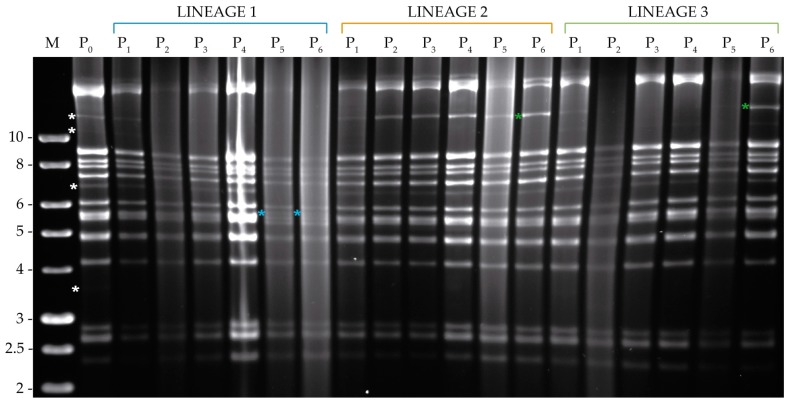
HindIII profiles of the genomic DNAs of the OBs obtained after serial passage of HearMNPV in the semi-permissive host, *S. littoralis*, from passage zero to six (P_0_–P_6_). M indicates 1 kb molecular size marker (Nippon) and fragment sizes are indicated on the left. White asterisks indicate HearMNPV-P_0_ submolar bands. The changes with respect to the P_0_ REN profile are marked by blue asterisks to indicate the appearance of a new fragment and green asterisks to indicate the intensification of an existing submolar fragment.

The HearMNPV-P_0_ inoculum showed four weak submolar HindIII fragments of ~3.6, 7, 11 and 12 kb (Figure 5). The 3.6 kb HindIII fragment totally disappeared after the first passage in all the lineages. The 7 kb HindIII fragment was not modified in lineages 2 and 3, but it disappeared in lineage 1 after the fifth passage. The 11 kb minor HindIII fragment disappeared after passage two in lineage 1 and after passage one in lineage 2, whereas in lineage 3, this fragment was maintained and slightly intensified at passage six. The 12 kb submolar HindIII fragment disappeared in lineage 1 after the second passage, whereas in lineage 3, it disappeared after two passages but reappeared at the final passage. In lineage 2 however, the 12 kb HindIII fragment increased in intensity until the final passage. Furthermore, in lineage 1 at passage five, a new HindIII fragment appeared of ~5.5 kb and was maintained at passage six (Figure 5).

#### 3.3.2. Analysis of Viral DNA Produced in *S. exigua*

**Figure 6:**
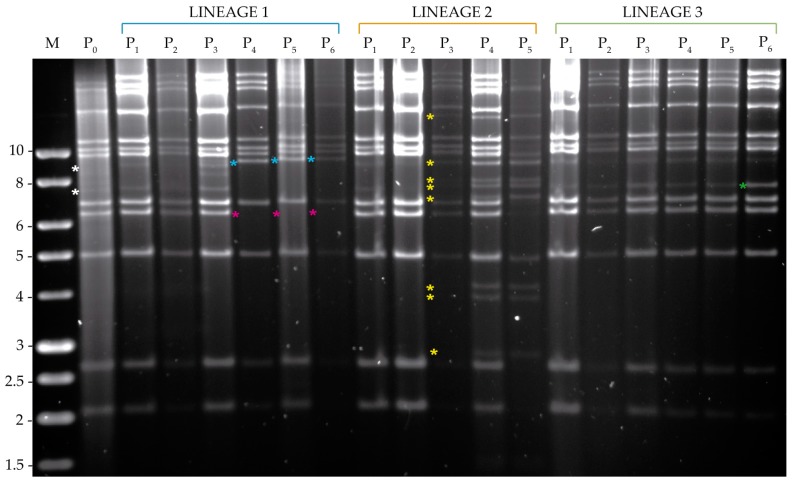
PstI restriction profiles of the genomic DNAs of OBs obtained after serial passage of MbMNPV in the permissive host, *S. exigua* from passage zero to six (P_0_–P_6_). M indicates 1 kb molecular size marker (Nippon) and fragment sizes are indicated on the left. White asterisks indicate MbMNPV-P_0_ submolar bands. The changes with respect to the P_0_ REN profile are marked by blue asterisks to indicate the appearance of a new fragment, magenta asterisks to indicate the disappearance of a band and green asterisks to indicate the intensification of an existing submolar fragment. Yellow asterisks indicate SeMNPV characteristic fragments from a SeMNPV contamination event.

The submolar PstI fragments of 7.5 and 9 Kb observed in MbMNPV-P_0_ tended to disappear in lineages 1 and 2, whereas they remained present through all the passage steps in lineage 3 (Figure 6). In lineage 1, a new PstI restriction fragment of ~7 kb appeared at passage four and a 6.3 kb fragment was lost. In lineage 2, faint new bands were observed after the third passage that increased in intensity after passage four. A marked change in the MbMNPV profile was observed after the fifth passage, which revealed that the new PstI fragments detected in passage three corresponded to the apparent activation of a previously inapparent SeMNPV infection [32]. An analysis by qPCR using specific primers confirmed the presence of 5.85 × 10^−1^ ng SeMNPV DNA/ng total DNA after the third passage in lineage 2. Passage four yielded 8.55 × 10^−1^ ng SeMNPV DNA/ng total DNA and at passage five, SeMNPV had totally displaced MbMNPV, as all the DNA present in the sample was SeMNPV genomic DNA in this lineage. Lineage 2 was then eliminated from the study and a new *S. exigua* population was established and checked to confirm the absence of a persistent SeMNPV infection. Passages four and five were repeated for MbMNPV lineages 1 and 3. The presence of SeMNPV was then quantified in passages one and two of lineage 2, passages three and six of the remaining lineages and the original MbMNPV-P_0_. In all cases, only trace quantities of SeMNPV DNA were present, with a mean (±SE) of 2.88 × 10^−6^ ± 1.50 × 10^−6^ ng SeMNPV DNA/ng total DNA (range 0.00–1.13 × 10^−5^ ng SeMNPV DNA/ng total DNA), which were consistent with the presence of very low level SeMNPV replication in the persistently infected *S. exigua* colony.

**Figure 7:**
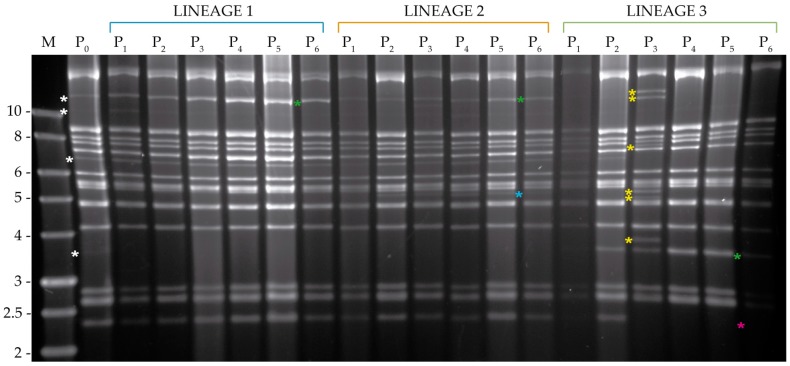
HindIII profiles of the genomic DNAs of OBs obtained after serial passages of HearMNPV in the permissive host, *S. exigua* from passage zero to six (P_0_–P_6_). M indicates 1 kb molecular size marker (Nippon) and fragment sizes are indicated on the left. White asterisks indicate HearMNPV-P_0_ submolar bands. The changes with respect to the P_0_ REN profile are marked by blue asterisks to indicate the appearance of a new fragment, magenta to indicate the disappearance of a band and green to indicate the intensification of an existing submolar fragment. Yellow asterisks indicate MbMNPV characteristic fragments from a cross-contamination event.

For HearMNPV, an intensification of the 12 kb submolar HindIII fragment present in HearMNPV-P_0_ was detected from the first passage to the last one in lineages 1 and 2 (Figure 7). In contrast, this HindIII fragment disappeared in lineage 3 from the second passage. Both the 11 kb and 7 kb submolar HindIII fragments were maintained in the three lineages, but the latter disappeared at the third passage in lineage 3. A 3.6 kb fragment totally disappeared in lineages 1 and 2, but markedly intensified in lineage 3 after the second passage. A new restriction HindIII fragment of approximately 5.1 kb was detected in lineage 2, whereas a 2.4 kb HindIII-fragment was lost in lineage 3. In addition in this lineage, six new HindIII fragments were detected at passage three. An examination of the HindIII-digested DNA of MbMNPV revealed that a cross-infection with this virus occurred in lineage 3. However, MbMNPV was not observed again after the fourth passage, so passages were completed for this lineage until the sixth passage. The presence of MbMNPV in these samples was quantified by qPCR at 0.34 ng MbMNPV DNA/ng of total DNA for passage three of lineage 3. The MbMNPV content diminished progressively in the following passages with values of 2.09 × 10^−4^, 1.18 × 10^−5^ and 9.58 × 10^−6^ ng MbMNPV DNA/ng total DNA for passages four, five and six, respectively. This is in agreement with the results of the REN study. SeMNPV was also quantified by qPCR in samples from passages three and six with an average of 3.84 × 10^−6^ ± 1.55 × 10^−6^ ng SeMNPV DNA/ng total DNA (range 0.00–1.10 × 10^−5^ ng SeMNPV DNA/ng total DNA). This is consistent with a very low level persistent infection in the *S. exigua* colony.

### 3.4. Insecticidal Properties of OBs after Passage in Semi-Permissive and Permissive Hosts

#### 3.4.1. Pathogenicity and Speed-of-Kill of OBs Following Passage in *S. littoralis*

The LC_50_ value of MbMNPV-P_0_ OBs varied from 2.6 × 10^7^ to 4.1 × 10^6^ OBs/mL in the semi-permissive host *S. littoralis*, depending on the bioassay, whereas LC_90_ values varied from 5.4 × 10^8^ to 1.3 × 10^8^ OBs/mL (Table 3). Similarly, the estimated LC_50_ value for HearMNPV-P_0_ OBs varied from 6.2 × 10^7^ to 1.9 × 10^7^ OBs/mL, whereas LC_90_ values were 27- to 89-fold higher. In all cases, the passage zero values were taken as the reference treatment (relative potency = 1) for bioassays of the pathogenicity of OBs from subsequent passages. After the third passage, all the lineages of MbMNPV OBs showed a 3- to 10-fold decrease in pathogenicity, whereas no marked changes were observed in the HearMNPV lineages compared to the P_0_ inoculum (Table 3).

A moderate decrease in pathogenicity of the MbMNPV lineage detected after passage three was maintained at passage six, with one exception in the LC_50_ value estimated for lineage 3. This was intermediate between the corresponding value for MbMNPV-P_0_ and the LC_50_ values estimated for lineages 1 and 2 (Table 3). No differences were observed in OB pathogenicity at the final passage when compared to the HearMNPV-P_0_ inoculum.

**Figure 8:**
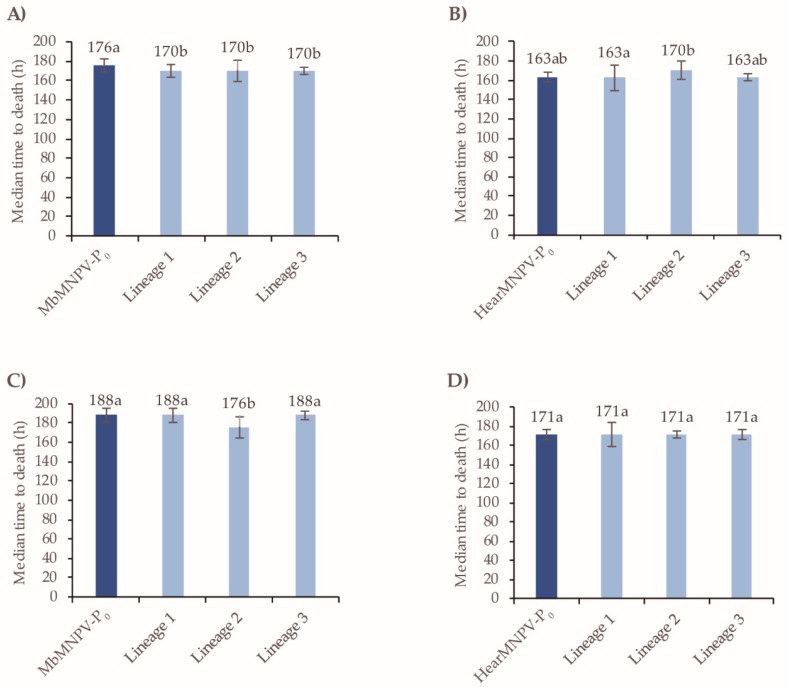
The speed-of-kill of MbMNPV and HearMNPV after passages three and six in *S. littoralis*. (**A**) MbMNPV-P_0_ and lineages after passage three, (**B**) HearMNPV-P_0_ and lineages after passage three, (**C**) MbMNPV-P_0_ and lineages after passage six, (**D**) HearMNPV-P_0_ and lineages after passage six. The median time to death values (MTD) were estimated by a Kapan-Meier analysis. The error bars correspond to 95% confidence limits. Values followed by different letters differ significantly (*p* < 0.05).

In terms of the speed-of-kill, a significant but small reduction of 6 h was observed in all three linages of MbMNPV after passage three compared to the MbMNPV-P_0_ inoculum (Log-Rank test: *χ*^2^ = 16.184, d.f. = 3, *p* = 0.001) (Figure 8A). These differences were not sustained in lineage 1 and 3 at the sixth passage, whereas lineage 2 remained slightly faster killing than the MbMNPV-P_0_ inoculum and the other lineages tested (Log-Rank test: χ^2^ = 14.207, d.f. = 3, *p* = 0.003) (Figure 8C). For HearMNPV, the speed-of-kill values did not change significantly at the third passage (Log-Rank test: χ^2^ = 5.291, d.f. = 3, *p* = 0.152), or the sixth passage compared to the HearMNPV-P_0_ inoculum (Log-Rank test: χ^2^ = 1.294, d.f. = 3, *p* = 0.731) (Figure 8B,D).

#### 3.4.2. Pathogenicity and Speed-of-Kill of OBs Following Passage in *S. exigua*

The LC_50_ values of MbMNPV-P_0_ OBs varied from 1.1 × 10^5^ to 5.3 × 10^4^ OBs/mL in the permissive host, whereas LC_90_ values varied from 1.6 × 10^6^ to 3.6 × 10^6^ OBs/mL (Table 4). For HearMNPV-P_0_ OBs, LC_50_ values varied from 6.2 × 10^4^ to 3.0 × 10^4^ OBs/mL, whereas the estimated LC_90_ values were between 8.9 × 10^5^ and 1.0 × 10^6^ OBs/mL. After the third passage, MbMNPV lineage 2 showed a 2- to 3-fold increase in pathogenicity, which seemed to be related to the SeMNPV contamination event detected in REN profiles at the third passage, which was confirmed by qPCR. This lineage was excluded from the study. Additionally, a 1.6-fold decrease in terms of LC_50_ was detected in lineage 3. Unexpectedly, a 6- to 17-fold reduction in pathogenicity was observed in HearMNPV lineage 3 compared to HearMNPV-P_0_, in which MbMNPV cross-infection was detected by REN and qPCR analysis (Table 4). After the sixth passage, no significant changes in pathogenicity were detected in either virus compared to MbMNPV-P_0_ and HearMNPV-P_0_.

**Figure 9:**
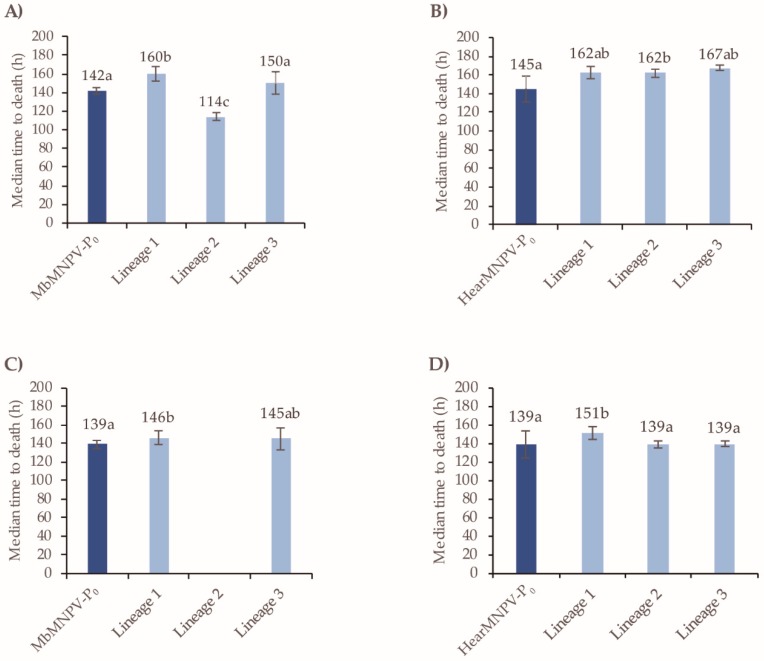
The speed-of-kill of MbMNPV and HearMNPV after passages three and six in *S. exigua*. (**A**) MbMNPV-P_0_ and lineages after passage three, (**B**) HearMNPV-P_0_ and lineages after passage three, (**C**) MbMNPV-P_0_ and lineages after passage six, (**D**) HearMNPV-P_0_ and lineages after passage six. The median time to death values (MTD) were estimated by Kapan-Meier analysis. The error bars correspond to 95% confidence limits. Values followed by different letters differ significantly (*p* < 0.05).

In terms of the speed-of-kill, after passage three, a significant increase in median time to death (MTD) of 18 h was observed in lineage 1 for MbMNPV (Log-Rank test: χ^2^ = 22.339; d.f. = 3, *p* < 0. 001) and of 17 h in lineage 2 for HearMNPV (Log-Rank test: χ^2^ = 6.227; d.f. = 3, *p* = 0.101) compared to the P_0_ inoculum (Figure 9A,B). However, a significant decrease in MTD of 28 h was detected in lineage 2 for MbMNPV. This is probably related to the SeMNPV infection that was detected. Following the sixth passage, an increase in MTD of 7 h was observed in lineage 1 for MbMNPV (Log-Rank test: χ^2^ = 14.054; d.f. = 2, *p* = 0.001) and of 12 h in lineage 1 for HearMNPV (Log-Rank test: χ^2^ = 23.394; d.f. = 3, *p* < 0.001) (Figure 9C,D).

## 4. Discussion

In the present study, MbMNPV and HearMNPV had a similar host range when used to inoculate heterologous lepidopteran species. The same lepidopteran species were considered as permissive, semi-permissive and non-permissive for both viruses. These species had been previously described as susceptible species for both viruses with the exception of *S. littoralis* [13,14,33,34]. In the present study, it was clear that *S. littoralis* was a semi-permissive host for both viruses, at lower inoculum concentrations than used in a previous study [13]. As restriction endonuclease profiles were checked in the OBs produced in each host, the authors are confident that this inconsistency was not the result of activation of latent virus infection in larvae of *S. littoralis* inoculated with heterologous viruses.

The OB concentration selected for the serial passage experiment was based on the desire to maintain a near-constant 25% prevalence of virus-induced mortality. Virus entry to midgut cells is the main barrier to the establishment of a successive infection [35], but once the secondary infection process has initiated, a low viral concentration may allow the production of a higher number or infection cycles within the host, which results in an increased viral yield [36,37]. In a previous study, low doses of the single-nucleocapsid HearNPV in *H. armigera* larvae resulted in an increase in genetic diversity [38]. Baillie and Bouwer [38] suggested that low doses may favor the production of a variety of novel genotypes that would not be detectable at high doses. At high multiplicities of infection, competition among genotypes may favor the proliferation of cooperative genotypes, or defective variants that exploit competent genotypes by complementation [38,39,40].

Despite the fact that all lineages were treated identically to increase the reproducibility of the technique, genetic analysis revealed differences in the REN profiles among the lineages in each host-virus combination, indicating that host-virus interactions did not follow a common genetic trajectory. This variability among lineages has been previously reported following serial passage of lineages established from a single common inoculum of HearNPV OBs [41]. Despite having a similar name, the single-nucleocapsid HearNPV is not closely related to the HearMNPV strain that we studied. Variation in host susceptibility, genotypic variant interactions within hosts, or transmission bottlenecks could be a possible explanation for the observed variation among different lineages [41]. Indeed, the insect midgut is a major site of selection for genotypes during transmission [42,43]. As such, it also represents a genetic bottleneck that is likely to strongly influence the diversity of founder genotypes in each host. Indeed, for nucleopolyhedroviruses, the number of virions that establish an infection has been estimated at approximately 1 - 2 virions in each insect for inoculum doses in which 20–30% of individuals become infected [44]. Although additional virions may achieve initial infection, processes such as clearance by host immune defenses, variation in the capacity to disperse to other host tissues during systemic infection, or the presence of defective genotypes is likely to further reduce the effective founder population [44]. In contrast, the presence of marked heterogeneity in host susceptibility can increase the effective founder population [45], as can the fact that multicapsid ODVs may contain multiple genotypic variants that are transmitted in groups [46]. In the present study, the authors considered this bottleneck as an opportunity for selection of the genotypes that were best adapted to each host during serial passage, as only the more infectious variants, or the genotypes that had replicated most efficiently and had reached the highest prevalence in each type of host, would be likely to establish a productive infection in the following passage step.

Changes in submolar bands were detected during serial passage in every host-virus system. Minor changes in restriction profiles may be associated with significant changes in the phenotypic characteristics of the virus [47,48,49]. These variations in the genetic structure of the wild-type isolates were likely due to a combination of founder effects during serial passage and virus interactions with the hosts or among genotypic variants [44]. Genetic diversity in wild-type nucleopolyhedrovirus populations is selectively advantageous and has clear ecological and evolutionary benefits to these viruses [50]. For example, mixtures of genotypes present in wild-type NPV isolates can increase the pathogen’s ability to develop productive infections over that of the component genotypic variants alone [51,52,53,54].

Modifications in REN profiles could also be due to the proliferation of minority genotypes or because new genotypes appeared. Genotypic variants can be generated by several mechanisms, such as recombination events, insertion or deletions of viral sequences, point mutations, horizontal gene transfer, as well as alterations in the relative abundance of genotype variants [18,20,21,55,56]. Further studies on the genetic structure of these isolates are required to elucidate the nature and genomic location of the genotypic variation detected in this study.

The REN profile variations detected in MbMNPV lineage 2 and HearMNPV lineage 3 after the third passage in the permissive host *S. exigua* were identified by qPCR as SeMNPV and a MbMNPV contamination event, respectively. The activation of persistent infections in lepidopteran larvae by alphabaculoviruses is well documented [57,58,59,60,61]. As SeMNPV totally displaced MbMNPV in the fifth passage in *S. exigua*, lineage 2 of MbMNPV had to be discarded and the other lineages were continued using a new population of *S. exigua*. After passage four, the presence of MbMNPV clearly decreased in HearMNPV lineage 3, so the presence of MbMNPV in the HearMNPV-inoculum was considered to be a transient contamination event and the experiment was continued. 

In the biological activity analyses, some small differences were observed among the lineages of each host-virus system. Although pathogenicity characteristics were similar among the lineages in each of the host-virus combinations, small differences in the speed-of-kill were detected in four out of the eight host-virus systems studied. Rovesti et al. [14] reported no differences in biological activity of isolates of these same viruses in their respective homologous and heterologous hosts, *M. brassicae* and *H. armigera*. In the present study, differences were observed between MbMNPV and HearMNPV in the insecticidal characteristics of the OBs following serial passage in *S. littoralis*. After the sixth passage, the insecticidal properties of HearMNPV did not show any alteration in pathogenicity or speed-of-kill compared to passage zero inoculum, whereas a significant decrease in MbMNPV pathogenicity was detected. The speed-of-kill of MbMNPV varied in each lineage but recovered the speed-of-kill of the original passage zero inoculum after six passages in two of the lineages. Several studies have demonstrated an increase in the insecticidal activity of a baculovirus in a particular semi-permissive host through serial passage [19,20,21,62], whereas others have reported no success in attempts at baculovirus adaptation to heterologous hosts [57,63,64]. The authors found no published evidence of deterioration in baculovirus insecticidal properties after serial passages in a heterologous host. Surprisingly, in view of the results obtained, lineage 2 of MbMNPV in *S. littoralis* showed an increase in its speed-of-kill, which was the only significant improvement in the insecticidal properties detected after the last passage in all the host-virus systems in this study.

In the permissive host *S. exigua*, the only alteration in insecticidal activity observed at the sixth passage compared to passage zero, was a significant decrease in the speed-of-kill in lineage 1 of both MbMNPV and HearMNPV, whereas the other lineages remained unchanged at the final passage. However, a significant improvement in biological activity, both in pathogenicity and speed-of-kill in lineage 2 of MbMNPV was detected after three passages, which was probably related to the presence of SeMNPV in the OB inoculum, as explained before. It is important to note that the activation of latent infections should not be excluded as a possible reason for marked improvements in insecticidal activity in serial passage host-adaptation studies in which no genetic characterization was performed on the progeny OBs [20]. As no improvements in the biological characteristics of the remaining lineages were observed, a biological effect of the minimal levels of SeMNPV present in the inocula was discarded. Furthermore, after the third passage, a significant decrease was observed in the pathogenicity of HearMNPV OBs from lineage 3 in *S. exigua* with respect to passage zero and the other lineages, which was associated with a transient contamination by MbMNPV that subsequently disappeared, possibly due to competitive exclusion by the HearMNPV population.

No clear correlation was observed between genetic and biological changes in any of the host-virus combinations analyzed. Indeed, lineages with REN profiles that seemed to be very similar elicited different biological responses. Others have reported changes in REN profiles with no marked effects on pathogenicity after passages in homologous and heterologous hosts [65,66].

Most studies on baculovirus adaptation to heterologous hosts have involved performing a high number of passages, often between 12 and 20. In some cases, an increase in the speed-of-kill and mortality has been detected after fewer than ten passages or even after a single passage [62,67]. However, the progeny OBs were not genetically characterized, therefore, a latent infection activation or a cross-contamination with another baculovirus cannot be precluded. In the present study, three passages in the heterologous hosts were sufficient to detect biological differences between MbMNPV and HearMNPV isolates. Optimization theory suggests that adaptation to a particular host is likely to constrain the pathogen’s ability to infect other host species. Consequently, pathogens that can infect multiple host species are likely to trade-off infectivity towards a particular host against host range. Due to this reason, they generally show lower levels of infectiousness, as the virus factors that promote infectivity cannot be optimized simultaneously for all possible hosts [68]. Therefore, the ability of broad host range pathogens to co-evolve with any one host is constrained.

This study highlighted similarities in the host range of HearMNPV and MbMNPV, but also indicated that HearMNPV appeared to be more phenotypically-stable than MbMNPV following serial passage in a semi-permissive host. However, host-baculovirus interactions are highly complex and differences among the three lineages in our study demonstrate that the process of adaptation to a novel host is far from predictable.

## Figures and Tables

**Table 1:** Primers used in this study.

Primers	Sequences	Amplification
Mb.F	5′- GGTATTAACGCCGCAACAAC-3′	MbMNPV, *bro-a1* (forward)
Mb.R	5′- TGGTTCTCCATGTTCGTCAA-3′	MbMNPV, *bro-a1* (reverse)
Se.F	5′- TCGTGCCGAAAGTGCCAGTA-3′	SeMNPV, *se28* (forward)
Se.R	5′- TTTGGCCAAAGTCGCGTTCG-3′	SeMNPV, *se28* (reverse)

**Table 2:** The host range of MbMNPV and HearMNPV in second instars of different lepidopteran pest species.

Host Species	MbMNPV	HearMNPV
*H. armigera*	permissive	permissive
*L. botrana*	non-permissive	non-permissive
*M. brassicae*	permissive	permissive
*O. nubilalis*	non-permissive	non-permissive
*S. exigua*	permissive	permissive
*S. littoralis*	semi-permissive	semi-permissive

**Table 3:** Pathogenicity of MbMNPV and HearMNPV after passages three and six in *S. littoralis*.

Virus	LC_50_ (OBs/mL)	Relative Potency	95% Confidence Limits		LC_90_ (OBs/mL)	Relative Potency	95% Confidence Limits	
			Low	High			Low	High
MbMNPV passage 3								
MbMNPV-P_0_	2.6 × 10^7^a	1			5.4 × 10^8^a	1		
Lineage 1	1.1 × 10^8^b	0.23	0.14	0.40	3.1 × 10^9^b	0.18	0.06	0.49
Lineage 2	7.6 × 10^7^b	0.34	0.19	0.58	4.1 × 10^9^b	0.13	0.04	0.42
Lineage 3	1.1 × 10^8^b	0.23	0.13	0.42	6.6 × 10^9^b	0.08	0.02	0.28
HearMNPV passage 3								
HearMNPV-P_0_	6.2 × 10^7^a	1			1.7 × 10^9^a	1		
Lineage 1	8.8 × 10^7^a	0.70	0.37	1.34	3.4 × 10^9^a	0.51	0.15	1.71
Lineage 2	7.8 × 10^7^a	0.80	0.43	1.46	1.8 × 10^9^a	0.99	0.33	2.93
Lineage 3	6.9 × 10^7^a	0.90	0.47	1.71	2.6 × 10^9^a	0.68	0.21	2.25
MbMNPV passage 6								
MbMNPV-P_0_	4.1 × 10^6^a	1			1.3 × 10^8^a	1		
Lineage 1	1.2 × 10^7^b	0.34	0.19	0.60	3.8 × 10^8^b	0.35	0.15	0.80
Lineage 2	1.1 × 10^7^b	0.37	0.20	0.68	6.2 × 10^8^b	0.22	0.09	0.54
Lineage 3	6.6 × 10^6^a,b	0.62	0.34	1.16	3.6 × 10^8^b	0.37	0.15	0.90
HearMNPV passage 6								
HearMNPV-P_0_	1.9 × 10^7^a	1			1.7 × 10^9^a	1		
Lineage 1	2.4 × 10^7^a	0.78	0.42	1.47	2.1 × 10^9^a	0.82	0.25	2.69
Lineage 2	2.2 × 10^7^a	0.87	0.47	1.60	1.5 × 10^9^a	1.21	0.39	3.78
Lineage 3	1.4 × 10^7^a	1.41	0.72	2.77	1.9 × 10^9^a	0.93	0.27	3.24

Probit regressions were fitted in POLO Plus (Leora). A test for non-parallelism was not significant for MbMNPV passage three (χ ^2^ = 7.57; d.f. = 3; *p*= 0.056), HearMNPV passage three (χ^2^ = 1.83; d.f. = 3; *p*= 0.608), MbMNPV passage six (χ ^2^ = 2.97; d.f. = 3; *p*= 0.396) and for HearMNPV passage six (χ ^2^ = 1.69; d.f. = 3; *p*= 0.639). The relative potencies were calculated as the ratio of effective concentrations relative to MbMNPV-P_0_ or HearMNPV-P_0_.

**Table 4:** The pathogenicity of MbMNPV and HearMNPV after three and six passages in *S. exigua*.

Virus	LC_50_ (OBs/mL)	Relative Potency	95% Confidence Limits		LC_90_ (OBs/mL)	Relative Potency	95% Confidence Limits	
			Low	High			Low	High
MbMNPV passage 3								
MbMNPV-P_0_	1.1 × 10^5^a	1			1.6 × 10^6^a	1		
Lineage 1	8.4 × 10^4^a,b,c	1.31	0.84	2.06	9.4 × 10^5^a,b	1.71	0.80	3.66
Lineage 2	5.9 × 10^4^b	1.86	1.20	2.88	5.4 × 10^5^b	2.98	1.45	6.15
Lineage 3	1.8 × 10^5^c	0.62	0.40	0.98	2.1 × 10^6^a,b	0.77	0.34	1.73
HearMNPV passage 3								
HearMNPV-P_0_	6.2 × 10^4^a	1			8.9 × 10^5^a	1		
Lineage 1	8.9 × 10^4^a	0.69	0.42	1.14	1.8 × 10^6^a	0.49	0.21	1.15
Lineage 2	6.5 × 10^4^a	0.95	0.59	1.53	9.1 × 10^5^a	0.98	0.45	2.12
Lineage 3	3.9 × 10^5^b	0.16	0.09	0.28	1.5 × 10^7^b	0.06	0.02	0.20
MbMNPV passage 6								
MbMNPV-P_0_	5.3 × 10^4^a	1			3.6 × 10^6^a	1		
Lineage 1	4.0 × 10^4^a	1.33	0.76	2.33	1.4 × 10^6^a	2.48	0.83	7.44
Lineage 2								
Lineage 3	4.7 × 10^4^a	1.14	0.64	2.06	2.7 × 10^6^a	1.35	0.42	4.39
HearMNPV passage 6								
HearMNPV-P_0_	3.0 × 10^4^a	1			1.0 × 10^6^a	1		
Lineage 1	2.3 × 10^4^a	1.30	0.74	2.29	1.4 × 10^6^a	0.74	0.27	2.06
Lineage 2	2.6 × 10^4^a	1.14	0.66	1.95	1.1 × 10^6^a	0.99	0.38	2.55
Lineage 3	2.5 × 10^4^a	1.22	0.72	2.07	7.8 × 10^5^a	1.34	0.54	3.33

The probit regressions were fitted in POLO Plus (Leora). A test for non-parallelism was not significant for MbMNPV passage three (χ^2^ = 2.78; d.f. = 3; *p*= 0.426), HearMNPV passage three (χ^2^ = 7.025; d.f. = 3; *p*= 0.071), MbMNPV passage six (χ^2^ = 2.11; d.f. = 2; *p*= 0.349) and for HearMNPV passage six (χ^2^ = 2.71; d.f. = 3; *p*= 0.439). The relative potencies were calculated as the ratio of effective concentrations relative to each MbMNPV-P_0_ or HearMNPV-P_0_.
